# Circulating Levels of the Heparan Sulfate Proteoglycan Syndecan-4 Positively Associate with Blood Pressure in Healthy Premenopausal Women

**DOI:** 10.3390/biom11030342

**Published:** 2021-02-24

**Authors:** Maria De Luca, David R. Bryan, Gary R. Hunter

**Affiliations:** Department of Nutrition Sciences, University of Alabama at Birmingham, Birmingham, AL 35294, USA; dbryan@uab.edu (D.R.B.); ghunter@uab.edu (G.R.H.)

**Keywords:** blood pressure, glycocalyx, body composition, arterial vasculature, catecholamines

## Abstract

Syndecans (SDCs) are transmembrane proteins that are present on most cell types where they play a role in multiple physiological processes, including cell–matrix adhesion and inflammation. Growing evidence suggests that elevated levels of both shed SDC1 and SDC4 are associated with hypertension and cardiovascular diseases, but their relationships with cardiovascular risk factors in healthy individuals are unknown. The primary objective of this study was to investigate whether serum levels of SDC4 and SDC1 were associated with body composition, hemodynamic parameters, pro-inflammatory cytokine concentrations, and urinary noradrenaline and dopamine levels in healthy women (17 African American and 20 European American) between the ages of 20 and 40 years old. Univariate analyses revealed only a significant (*p* < 0.05) inverse correlation between serum SDC1 and body fat percentage. On the other hand, serum SDC4 was positively correlated with systolic blood pressure, diastolic blood pressure, and urinary levels of noradrenaline and dopamine. Serum SDC4 was also a significant predictor of systolic blood pressure in a multivariate regression model that included fat-free mass and urinary dopamine levels as significant independent variables. The result did not change even adjusting for race. Our findings indicate that SDC4 has an important role in the physiological regulation of blood pressure.

## 1. Introduction

Syndecans (SDCs) are type-I transmembrane glycoproteins belonging to the family of heparan sulfate proteoglycans that also include cell-surface glycosylphosphatidylinositol-anchored proteins (glypicans) and secreted proteoglycans found in basement membranes (agrin, collagen XVIII, and perlecan) [1]. Four SDC proteins, SDC1 to 4, are present in vertebrates, with SDC4 being distributed ubiquitously and the others having a tissue-specific expression pattern [2]. All SDCs are characterized by an extracellular domain (ectodomain) with attachment sites for glycosaminoglycan (GAG) chains (e.g., heparan sulfate and chondroitin sulfate) that mediate interactions with a wide array of ligands, such as extracellular matrix components, soluble growth factors, morphogens, chemokines, and cytokines [1]. The ectodomain of all SDCs is constitutively released from the cell surface by proteolytic cleavage in a process known as ectodomain shedding that is mediated by matrix metalloproteinases [3]. SDC shedding is a highly regulated process that can be accelerated by various physiological agents through the activation of intracellular signaling cascades, such as the mitogen-activated protein kinase/extracellular signal-regulated kinase and c-Jun N-terminal kinase pathways [4]. Once released from the cell surface, the ectodomains may act as paracrine or autocrine effectors or compete with cell surface receptors for the same ligand [5]. SDCs also contain a highly conserved transmembrane domain and a short cytoplasmic tail [1]. It is through their cytoplasmic domain, which includes binding sites for cytoskeletal proteins and protein kinases, that members of the SDC family can control cell behavior in synergy with the integrin-mediated signaling and/or independently of integrins [6,7]. Moreover, the cytoplasmic domain is essential in the regulation of SDC shedding [8]. 

In recent years, it has become clear that the endothelial glycocalyx (eGC) surrounding the surface of endothelial cells is a key determinant of vascular permeability, inflammation, and elasticity [9]. The glycocalyx, also known as pericellular matrix, is a meshwork layer of highly charged glycolipids and glycoproteins coating the outside of most cell types [10]. Among the major components of the glycocalyx, there are SDC1 and SDC4, which participate as mechanosensors in the shear-induced nitric oxide production by endothelial cells [11,12,13]. A growing body of evidence indicates that SDC1 is released into the circulation in response to endothelial injury [14,15] and elevated levels of serum SDC1 have been detected in patients with severe sepsis [16] and have been associated with adverse clinical outcomes in patients with ischemic heart failure [17]. More recent studies have also shown positive associations of SDC4 levels with coronary heart disease in women [18] and with resistant hypertension [19]. Together, these findings suggest that circulating SDC1 and SDC4 levels could be useful predictive markers of eGC damage in selected pathologies [17,18,20]. However, given the complexity of SDC biology [21], more work is needed to better understand the importance of soluble SDCs as predictors of vascular disease [17]. For instance, there is still little information on the inter-individual variability of circulating SDC4 and SDC1 levels in healthy populations, even if these variations could have an impact on vascular inflammation and elasticity as well as adiposity, as suggested by studies in model systems [22,23,24]. This is particularly true when it comes to women whose risk to develop hypertension is often underestimated and undiagnosed [25]. To this end, the primary objective of this study was to investigate the associations of serum SDC4 and SDC1 levels with body composition phenotypes, hemodynamic parameters, circulating pro-inflammatory cytokines, and 24-h urine levels of catecholamines in a healthy cohort of African American (AA) and European American (EA) premenopausal women. It is well-established that AAs on average have higher blood pressure levels than EAs [26]. As such, a secondary objective of this study was to determine whether variability in SDC4 and/or SDC1 might contribute to the racial disparity in blood pressure levels. Finally, given that serum SDC4 levels have been positively associated with hypertension [19] and exercise training has been shown to have beneficial effects on blood pressure arterial stiffness [27], another aim was to investigate the effect of long-term aerobic exercise on the abundance of circulating SDC4. 

## 2. Materials and Methods

### 2.1. Study Participants and Design 

The objectives of our investigation were assessed in a cohort of 37 women between the ages of 20 and 40 years. Subjects were previously enrolled to participate in an intervention study specifically designed to determine the effect of exercise on blood pressure. The design of the intervention study, which initially included 22 women, is extensively described by Carter et al. [28]. Briefly, women were included in the study if they reported normal menstrual cycles and were not taking oral contraceptives or any medications. Additionally, they were (i) normotensive; (ii) normoglycemic, as evaluated by postprandial glucose response to a 75-g oral glucose tolerance test; (iii) non-smoker; and (iv) sedentary. After data baseline collection, participants began supervised aerobic exercise training on a stationary cycle ergometer held three days per week for 16 weeks. Participants were evaluated again in random order under standardized conditions at weeks 8, 12, and 16. Participants abstained from any exercise 48 h prior to testing for the purpose of attenuating the acute effects of exercise. 

The baseline data of the variables described in the following sections were used in this investigation. Serum SDC4 and SDC1 levels were measured in blood samples collected at baseline. To examine the effect of exercise on serum SDC4 levels, SDC4 was measured at baseline and at the end of weeks 8, 12, and 16 of the exercise regime.

### 2.2. Body Composition

Body weight was measured with a Detecto eye-level mechanical weigh beam scale. Total and regional body composition (i.e., fat mass (FM) and fat-free mass (FFM)) was determined by dual-energy X-ray absorptiometry (DXA; iDXA, GE-Lunar, Madison, WI, USA) as reported in [28].

### 2.3. Blood Pressure and Arterial Elasticity

Systolic blood pressure (SBP), diastolic blood pressure (DBP), large artery elasticity (LAE), small artery elasticity (SAE), systemic vascular resistance (SVR), and estimated cardiac output (ECO) were measured at 7:00AM using non-invasive, local pulse contour analysis (HDI/Pulse Wave TM CR-2000, Hypertension Diagnostics Inc., Eagan, MN, USA). Pulse contour analyses from the radial artery are based on a modified Windkessel model that allows for the evaluation of microcirculatory vessels and is comprehensively described in [29]. Briefly, participants were seated and remained quiet during the testing procedure. An adult-sized blood pressure cuff was placed around the non-dominant arm. After palpating the radial pulse, a solid-state pressure transducer was fastened over the radial artery of the dominant arm. At this point, the sensor was adjusted as needed to achieve the highest relative signal strength, and arterial elasticity was determined by gathering and analyzing 30-s analog tracings of the radial waveform digitized at 200 samples/sec. Then, a beat-marking algorithm was used to assess the beginning systole, peak systole, onset of diastole, and end diastole for each beat during the 30-s period. The average beat was finally incorporated into a parameter-estimating algorithm, and the modified Windkessel model was used to determine LAE, SAE, SVR, and ECO [29]. All assessments were performed in triplicate and averaged for analysis.

### 2.4. Fractionated Catecholamines

As reported in [28], norepinephrine (NE) and dopamine (DA) were measured by high-performance liquid chromatography in 24-h urine samples treated with hydrochloric acid and glutathione and stored at −20 °C until assayed.

### 2.5. Laboratory Analyses

Blood samples were obtained following an overnight fast. Serum SDCs and pro-inflammatory cytokine concentrations were assessed using high-sensitivity enzyme-linked immunosorbent assay (ELISA) kits, following the manufacturer’s instructions for SDC1 (Human CD138 ELISA kit, Diaclone SAS, Besancon, France), SDC4 (Human Syndecan-4 Assay kit, Immuno-Biological Laboratories, Minneapolis, MN, USA), tumor necrosis factor-α (TNF-α) (Quantikine HSTA00C, R&D Systems, Minneapolis, MN, USA), interleukin-6 (IL-6) (Quantikine HSTA00C, R&D Systems, Minneapolis, MN, USA), and C-reactive protein (CRP) (CRP ELISA kit, ALPCO, Windham, NY, USA). All samples were analyzed in duplicate. 

### 2.6. Statistical Analysis

Statistical analysis was performed using SAS 9.4 (SAS Institute Inc, Cary, NC, USA). Descriptive characteristics are reported as means and standard errors. Data from subjects stratified by race were compared by an independent group Student’s *t*-test. Correlations between study variables were investigated by bivariate Spearman correlation statistics using the pooled data. To understand the relationship between SDC4 and blood pressure, multiple linear regression analyses were developed for SBP and DBP. Due to limitations of observations to variable ratio, three separate multiple regression models were run. Changes in body composition characteristics and intrarenal dopamine are associated with hypertension [30,31]. Moreover, FFM, the more metabolically active body portion, has been reported to have a more predominant effect on the human brain [32]. As such, body fat percentage (BF%) and FFM were selected as independent variables in model 1 and FFM and urinary DA levels were selected as independent variables in model 2. Given that arterial stiffness has been suggested to precede the development of high blood pressure [33], LAE and SAE were instead included in model 3. All models were also adjusted for race. The Shapiro–Wilk test was used to check the assumption of normality for outcomes of interests before performing regression analyses. Two (time) by two (race) analysis of variance (ANOVA) with repeated measures for treatment was used to compare differences in serum SDC4 levels at baseline and after 8–16 weeks of aerobic training. *p* values of <0.05 were considered statistically significant.

## 3. Results

### 3.1. Premenopausal AA Women Have Higher Abundance of Circulating SDC4 than EA Women.

The 37 subjects included 17 AA and 20 EA premenopausal women, with a mean age of 32.00 ± 0.93 years. Body composition, hemodynamic, pro-inflammatory cytokine, and catecholamine measures of the AA and EA women are reported in Table 1. 

**Figure 1:**
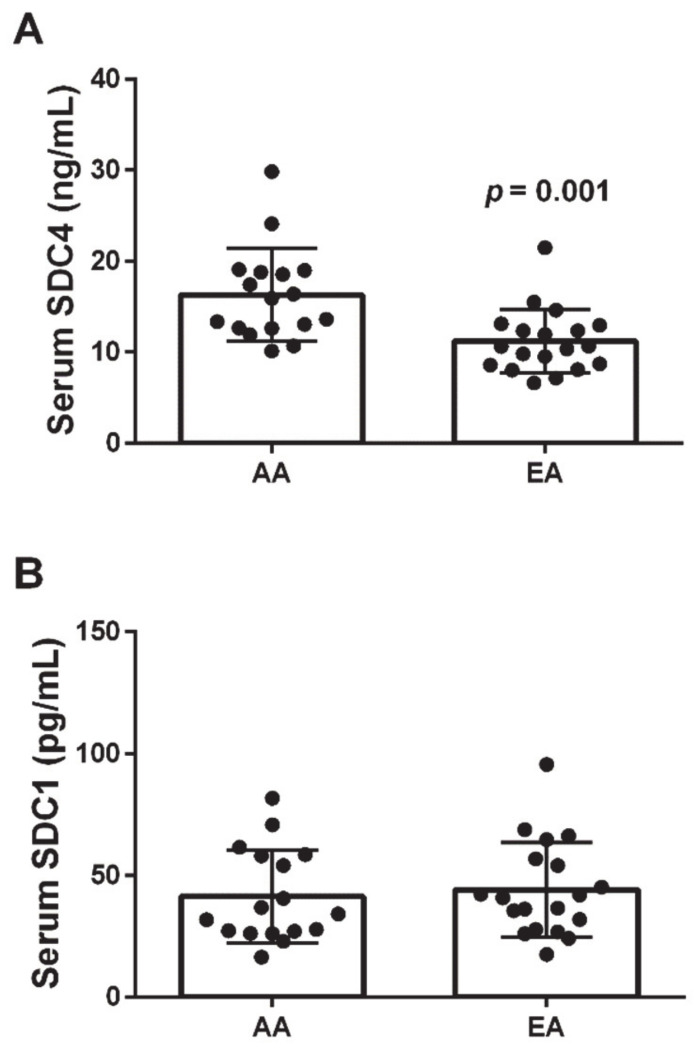
Race-specific differences in serum SDC4 concentrations across healthy premenopausal women. African American (AA) women have significantly higher levels of circulating SDC4 than European American (EA) women (**panel A**), but similar levels of circulating SDC1 (**panel B**).

There were no differences between AA and EA in body fat (e.g., FM and BF%), arterial elasticity, SVR, and circulating markers of inflammation. However, significant (*p* < 0.05) differences were observed for FFM, SBP, DBP, and levels of NE and DA, with AA women having on average higher levels of each variable. As shown in Figure 1, AA women also displayed significantly higher levels of circulating SDC4 than EA women (Figure 1A), but this disparity was not accompanied by a corresponding difference in SDC1 levels (Figure 1B). 

### 3.2. An Independent Relationship Exists between Serum SDC4 and Blood Pressure in Healthy Premenopausal Women

Table 2 reports the results of the univariate analysis used to identify factors associated with serum SCD4 and SDC1 in our cohort of healthy premenopausal women. 

**Figure 2:**
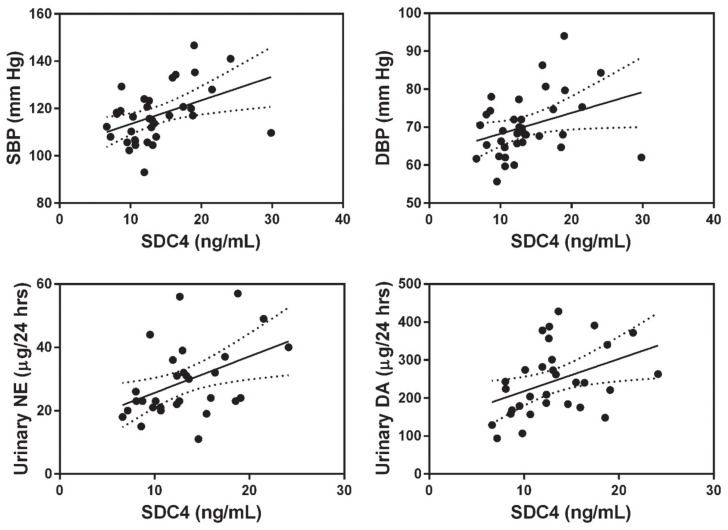
Serum levels of syndecan-4 (SDC4) are positively associated with blood pressure and urinary catecholamine concentrations. Scatterplots of the association between SDC4 (*n* = 37) and systolic blood pressure (SBP) (*n* = 37), diastolic blood pressure (DBP) (*n* = 37), urinary norepinephrine (NE) levels (*n* = 33), and urinary dopamine (DA) levels (*n* = 33). Shown in the plots is the 95% confidence band of the best-fit line.

Contrary to our expectations, there were no significant associations of circulating levels of SDC4 or SDC1 with either arterial elasticity parameters or pro-inflammatory cytokines. However, our analysis demonstrated statistically significant positive correlations of serum SDC4 levels with SBP, DBP, NE, and DA. The associations between SDC4 and these variables are depicted in Figure 2.

### 3.3. The Association between Circulating Levels of SDC4 and Blood Pressure is Independent of Differences in Body Composition, Urinary Dopamine, and Race

Multiple linear regression analysis was performed to understand the relationship of SDC4 with blood pressure, and the results are reported in Table 3. The analysis revealed that serum SDC4 remained significantly associated with SBP levels after adjusting for body composition variables (model 1) or for FFM and urinary DA levels (model 2). According to model 2, 56% of the variation in SBP in our cohort of healthy premenopausal women is explained by their levels of SDC4, FFM, and urinary DA levels. Notably, the results did not change even if race was included in the models. However, the relationship between SDC4 and SBP no longer existed when LAE, SAE, and race were included in the model (model 3). Overall, these findings suggest that the relationship between SDC4 and SBP is independent of differences in FFM and urinary DA levels and that the effect of race on SBP is blunted when these variables are included in the model. 

The involvement of SDC4 in the relationship between race and blood pressure was confirmed by multivariate regression models for DBP. As reported in Table 3, the association between serum SDC4 and DBP remained significant after adjusting for FFM, urinary DA levels, and race (model 2), with none of these variables showing a significant relationship with DBP. 

### 3.4. Long-Term Aerobic Exercise Does Not Impact the Abundance of Circulating SDC4

**Figure 3:**
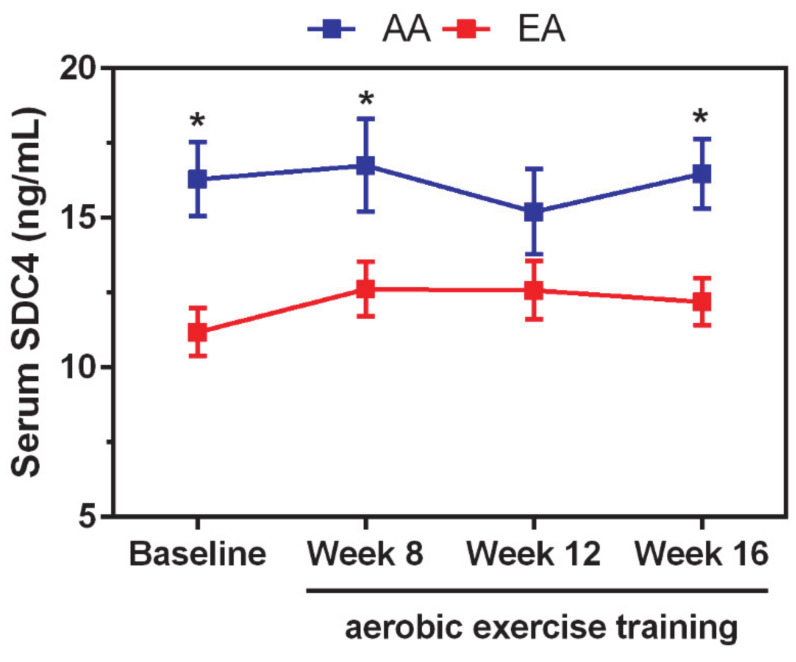
Serum SDC4 levels are not affected by long-term aerobic exercise training. Compared to European Americans (EAs), African American (AA) women showed statistically significantly higher levels of SDC4 at baseline and after 8 and 16 weeks of aerobic exercise regime. However, the levels of SDC4 did not change during the training. Error bars represent standard errors. Significant comparisons were determined by post hoc Tukey’s test at * *p* < 0.05.

A two-way repeated measures ANOVA performed to investigate whether exercise had an impact on serum SDC4 levels revealed a significant effect of race (*F*_1,135_ = 25.08; *p* < 0.0001), confirming the pre-training observation. As shown in Figure 3, after adjustment for multiple comparisons, AA women showed significantly higher levels of SDC4 than EA women not only at baseline but also after 8 weeks and 16 weeks of aerobic exercise. However, no treatment effect (*F*_3,135_ = 0.28; *p* = 0.8422) or treatment-by-race interaction (*F*_3,135_ = 0.41; *p* = 0.7483) were observed, indicating that long-term aerobic exercise did not change the levels of shed SDC4.

## 4. Discussion

The primary finding of the present study is that elevated levels of serum SDC4 significantly correlate with increased SBP and DBP as well as 24-h urine NE and DA in a cohort of healthy premenopausal women. Although the correlations observed in our study are in the low to moderate range, the findings corroborate the involvement of SDC4 in blood pressure regulation as previously seen by Lipphard and colleagues [19], who revealed that patients with resistant hypertension had significantly higher serum SDC4 levels than healthy controls. Moreover, the relationship between SBP, serum SDC4, and catecholamine levels is in agreement with results from the same study, which further showed that β-blockers had lowering effects on serum SDC4 levels of hypertensive patients [19]. It is well-established that catecholamines regulate blood pressure [34]. In high concentrations, catecholamines can also directly damage the endothelium most likely by enhancing eGC degradation [35,36,37]. For instance, circulating NE and epinephrine were found significantly correlated with plasma SDC1 in patients with ST elevation myocardial infarction, with the highest levels of epinephrine and SDC1 in patients with shock prior to primary percutaneous coronary intervention [36]. Our study did not show a correlation of serum SDC1 with catecholamines or blood pressure, suggesting that eGC damage is likely not responsible for the elevated serum SDC4 in the women who displayed the higher levels of blood pressure. This is further corroborated by the fact that there was no correlation between SDC4 and circulating pro-inflammatory cytokines, which are an end product of damaged glycocalyx [38]. 

Endothelial functions, including angiogenesis and the regulation of vascular wall homeostasis and inflammation, are influenced by glycocalyx-mediated mechanotransduction [39]. Studies in mice have shown that SDC4 is involved in the endothelial cell sensing of blood flow direction and is a potent anti-atherosclerotic molecule [11]. Based on these observations, serum levels of SDC4 were expected to be associated with parameters of vascular elasticity and SVR. However, this appears not to be the case in our cohort of healthy premenopausal women, suggesting an alternative mechanism of action. Myocardial fibrosis is a critical component of cardiac remodeling, and as argued by Tomek and Bub [40], pressure overload-induced cardiac fibrosis and elevated activity of the cardiac sympathetic nervous system (which provides inotropic support) may be compensatory and adaptive mechanisms occurring to maintain cardiovascular homeostasis. SDC4 has been reported to protect the pressure-overloaded heart of mice from the profibrotic effects of the matricellular protein osteopontin [41]; this protection is lost when the SCD4 ectodomain is shed [41]. Thus, it is plausible that increases in blood pressure may lead to SDC4 shedding due to its involvement in cardiac remodeling. This is corroborated by our finding of significant correlations between serum SDC4 and urinary catecholamines and a trend toward significance (*p* = 0.1) for the association between SDC4 and ECO (Table 2). Moreover, large arterial stiffness has been reported to contribute to left ventricular remodeling in hypertension [42], which might explain why the relationship between SDC4 and SBP no longer existed when LAE was included in the multivariate regression model. However, additional studies are needed to validate our hypothesis. 

The benefits of exercise for arterial stiffness are recognized [27]. Our study showed no effect of long-term aerobic exercise training on circulating levels of SDC4, corroborating the idea that the association between SDC4 and blood pressure is most likely not explained by a direct role of SDC4 in endothelial glycocalyx. Moreover, our finding echoes previous work showing no effect of 12-week aerobic exercise training on serum SDC4 levels in sedentary men [43].

Racial differences in blood pressure levels have been reported at all ages [44,45,46]. Consistently, AA women in our study, on average, had significantly higher SBP and DBP than EAs. Several factors have been proposed as contributors to the disparity, including body mass [26]. In our cohort of premenopausal women, there were no differences in FM and BF% between AAs and EAs, but a significant difference was observed in FFM, with AA women having, on average, more FFM. Additionally, AAs had higher levels of serum SDC4 and 24-h urine NE and DA than EAs. Notably, the inclusion of SDC4, FFM, and DA in our model for the estimation of SBP variance blunted the effect of race, suggesting that these variables could be confounders of racial disparity in SBP among normotensive women. DA and DA receptors are key players in the control of SBP, in part, by regulating renal sodium excretion, diuresis, and natriuresis and thereby circulatory volume [30]. The dysregulation of the intrarenal dopamine system can lead to the development of salt sensitive hypertension [30], and racial/ethnic differences in intake and handling of sodium have been reported in female adolescents, with AA girls having increased renal reabsorption of sodium under conditions of increased dietary salt intake [47]. As such, our finding supports the idea that underlying mechanisms that promote salt sensitivity are contributing factors associated with racial disparities in SBP variance and hypertension [26]. In this regard, it is important to point out that salt sensitivity and sodium homeostasis are impacted not only by kidney malfunction but also by changes in the vascular eGC layer [48]. Growing evidence suggests that the negatively charged heparan sulfate GAG chains covalently attached to the ectodomain of SDCs can bind and inactivate sodium, thereby acting as an intravascular sodium buffer for circulating sodium [49]. Thus, it is also possible that the association between circulating SDC4 levels and blood pressure is due to the involvement of SDC4 in the sodium buffer function of the vascular eGC layer.

Although no correlation between SDC1 and hemodynamic parameters was observed, our study also revealed a negative correlation between serum SDC1 and BF%. This result agrees with growing evidence in model systems suggesting that SDCs play a key role in adipose tissue biology [24,50,51,52]. For instance, previous studies performed in 3T3-L1 mouse adipocytes demonstrated that SDC1 is expressed in mature white adipocytes and its synthesis as well as the proteolytic shedding of its ectodomain are regulated by insulin [24]. Additionally, it was revealed that shed adipocyte SDC1 associates with endothelial-bond lipoprotein lipase, stabilizes its activity, and translocates it to the adipocytes [24]. Little is still known about SDC1 and adipose tissue in humans, but our finding indicates that more research should be conducted in this area. 

This study has some limitations: first, the small sample size and cross-sectional design. Second, since the study was not designed specifically to investigate the link between SDCs and blood pressure, our cohort contained only women, and it is possible that findings may not generalize to men. Estrogen promotes endothelial-derived nitric oxide production and could be a protective factor in the vascular response to challenges in premenopausal women [53]. Third, 24-h dietary salt intake and urinary sodium excretion data were not collected. Thus, the idea that variability in dietary sodium intake might underlie the link between SDC4 and SBP could not be tested. 

## 5. Conclusions

In conclusion, this study has shown that whereas serum SDC1 levels correlate only with adiposity in a cohort of healthy AA and EA premenopausal women, elevated levels of serum SDC4 associate with increased SBP, DBP, and 24-h urine levels of NE and DA. Since neither serum levels of SDC1 or SDC4 were significantly associated with vascular elasticity parameters or pro-inflammatory cytokines, our findings suggest that eGC damage may not be responsible for the relationship between serum SDC4 and blood pressure. Furthermore, serum SDC4 was a significant predictor of SBP in a multivariate regression model that also included FFM and urinary DA levels as significant independent variables. Notably, despite a statistically significant difference observed between AA and EA women in blood pressure, the relationship between race and SBP was no longer significant after adjusting for those variables. Finally, in line with previous work in sedentary men [43], our study showed that the levels of SDC4 did not change during 16-week aerobic exercise training. Together, these findings motivate additional work in large cohorts to pinpoint the underlying mechanism of the physiological relationship between SDC4 and blood pressure.

## Figures and Tables

**Table 1:** Descriptive statistics of study participants according to race.

Variables	African American (*n* =17)	European American (*n* = 20)	*p*-Values
Age (years)	32.44 ± 1.30	31.62 ± 1.39	0.568
FM (kg)	31.86 ± 2.21	25.77 ± 2.35	0.133
BF%	39.05 ± 1.37	36.21 ± 1.79	0.230
FFM (kg)	47.73 ± 1.61	42.04 ± 0.91	0.004
SBP (mm Hg)	121.89 ± 2.97	112.19 ± 2.14	0.010
DBP (mm Hg)	73.41 ± 2.20	67.39 ± 1.44	0.023
LAE (mL/mm Hg·100)	17.10 ± 1.46	15.80 ± 0.99	0.352
SAE (mL/mm Hg·100)	6.89 ± 0.58	7.73 ± 0.53	0.355
SVR (dyne/s/cm^−5^)	1331.05 ± 74.14	1324.75 ± 32.23	0.936
ECO (L/min)	5.60 ± 0.19	5.14 ± 0.14	0.059
Serum CRP (mg/L)	2.09 ± 0.39	2.51 ± 0.72	0.607
Serum IL-6 (pg/mL)	1.70 ± 0.65	2.79 ± 1.43	0.498
Serum TNF-α (pg/mL)	3.05 ± 0.33	3.14 ± 0.25	0.774
Urinary NE (µg/24 h)^a^	33.43 ± 3.01	25.22 ± 2.46	0.041
Urinary DA (µg/24 h)^a^	299.71 ± 22.06	205.28 ± 18.72	0.007

Values are presented as means ± standard error. Abbreviations: FM, fat mass; BF%, body fat percentage; FFM, fat-free mass; SBP, systolic blood pressure; DBP, diastolic blood pressure; LAE, large arterial elasticity; SAE, small arterial elasticity; SVR, systemic vascular resistance; ECO, estimated cardiac output; CRP, C-reactive protein; IL-6, interleukin-6; TNF-α, tumor necrosis-factor- α; NE, norepinephrine; DA, dopamine. Significant (*p* < 0.05) comparisons are indicated in bold; ^a^ denotes African Americans *n*=15 and European Americans *n* = 18.

**Table 2:** Correlations between serum levels of SDCs and study variables.

	Serum SDC4		Serum SDC1	
	*r*	*p*-Value	*r*	*p*-Value
BF%	0.2585	0.1280	−0.4135	**0.0122**
FFM	0.2201	0.1971	−0.0967	0.5749
SBP	0.4202	**0.0120**	0.1921	0.2688
DBP	0.3611	**0.0331**	0.2980	0.0821
LAE	−0.1720	0.3232	−0.1915	0.2705
SAE	−0.2108	0.2242	0.1606	0.3568
SVR	−0.0171	0.9223	0.2498	0.1479
ECO	0.2785	0.1052	−0.1423	0.4149
Serum CRP	−0.2154	0.2072	−0.0932	0.5888
Serum IL-6	−0.1130	0.5118	−0.1333	0.4383
Serum TNF-α	−0.1482	0.3884	−0.2150	0.2080
Urinary NE	0.4494	**0.0112**	0.1462	0.4326
Urinary DA	0.4341	**0.0147**	0.0782	0.6757

Abbreviations: BF%, body fat percentage; FFM, fat-free mass; SBP, systolic blood pressure; DBP, diastolic blood pressure; LAE, large arterial elasticity; SAE, small arterial elasticity; SVR, systemic vascular resistance; ECO, estimated cardiac output; CRP, C-reactive protein; IL-6, interleukin-6; TNF-α, tumor necrosis-factor- α; NE, norepinephrine; DA, dopamine. Significant (*p* < 0.05) associations are indicated in bold.

**Table 3:** Multiple linear regression models for associations of SDC4 levels with SBP and DBP.

		Independent Variables	Standardized Coefficient (β)	*p*-Values
SBP	Model 1 *p* = 0.0096 R^2^ = 0.35	SDC4	0.8828	0.0408
		BF%	−0.3368	0.2068
		FFM	0.7176	0.0389
		Race	1.6507	0.7187
	Model 2 *p* = 0.0003 R^2^ = 0.56	SDC4	1.5683	0.0008
		FFM	0.7215	0.0155
		DA	−0.0482	0.0149
		Race	1.2001	0.7683
	Model 3 *p* = 0.0007 R^2^ = 0.46	SDC4	0.1455	0.7183
		LAE	−1.3221	0.0016
		SAE	0.4358	0.5577
		Race	12.0860	0.0070
DBP	Model 1 *p* = 0.0113 R^2^ = 0.34	SDC4	0.4898	0.1020
		BF%	−0.4648	0.0168
		FFM	0.3796	0.1146
		Race	2.8209	0.3840
	Model 2 *p* = 0.0306 R^2^ = 0.34	SDC4	0.7201	0.0445
		FFM	0.2786	0.2383
		DA	−0.0137	0.3795
		Race	2.2456	0.5082
	Model 3 *p* = 0.0022 R^2^ = 0.42	SDC4	−0.1048	0.7207
		LAE	−0.9781	0.0013
		SAE	0.6203	0.2547
		Race	9.4848	0.0039

Abbreviations: SBP, systolic blood pressure; DBP, diastolic blood pressure; SDC4, syndecan-4; BF%, body fat percentage; FFM, fat-free mass; DA, dopamine; LAE, large arterial elasticity; SAE, small arterial elasticity. Significant (*p* < 0.05) effects are indicated in bold.

## Data Availability

The data presented in this study are available on request from the corresponding author. The data are not publicly available due to participants’ privacy protection.
